# Supplementary material for: Comparison of bone mineral density of runners with inactive males: A cross-sectional 4HAIE study
Source: PLoS One. 2024 Aug 9;19(8):e0306715. doi: 10.1371/journal.pone.0306715 (PMC11315333; doi:10.1371/journal.pone.0306715)
Supplement: S1 File — (ZIP) [file pone.0306715.s001.zip › S1_File_BMD_Mileage_Threshold.docx]

Partial analysis has been performed to provide additional data on the possible beneficial threshold of running and BMD. For this analysis we used information about running mileage and frequency from the surveys.

Three partial analyses were performed. For the first analysis the runners were divided into 9 sub-groups (inspired by MacDougall et al. [1]): 10-15 km/week, 16-20 km/week, 21-25 km/week, 26-30 km/week, 31-35km/week, 36-40 km/week, 41-45 km/week, 46-50 km/week, ≥51 km/week. Additionally, a pairwise comparison of different running mileage groups was conducted. Results of this analysis can be seen in Fig 2. For the second analysis the runners were divided into 2 sub-groups: <30 km/ week and ≥30 km/week. For the third analysis (based on the findings from Barrack et al. [2]) the runners were divided into 2 sub-groups: <50 km/ week and ≥50 km/week. To compare BMD of AR in different running mileage groups and eliminate confounding variables, ANCOVA with Bonferroni correction was used in all analyses. Confounding variables for all analyses were age, mass, height, BMI, fat mass, and lean mass. All three analyses found no significant difference in BMD between groups at every measured site (p>0.05). Results of the second and third analysis can be seen in Table 5 and 6, respectively.

These analyses were the only three methods of group separation we deemed reasonable. Higher mileage (90-100 km/week as stated by previous studies [3–5]) groups were not possible to use and compare.

Please insert Figure 2 here

**Fig 2.** Comparison bone mineral density of active runners in different running mileage group

**Table 5.** Comparison bone mineral density of active runners in different running mileage group (<30 km/week and ≥30 km/week)

| **Variable** | **<30 (*n* *=* 196)** | **≥30 (*n* = 131)** | ***F*** | **Sig.** |
| --- | --- | --- | --- | --- |
| **WB BMD (g/cm^2^)** | 1.16 ± 0.08 | 1.16 ± 0.08 | 0.182 | 0.670 |
| **Spine BMD (g/cm^2^)** | 1.08 ± 0.13 | 1.05 ± 0.13 | 1.324 | 0.251 |
| **LS BMD (g/cm^2^)** | 1.06 ± 0.13 | 1.04 ± 0.13 | 0.766 | 0.382 |
| **Dominant leg BMD (g/cm^2^)** | 1.25 ± 0.09 | 1.26 ± 0.08 | 0.703 | 0.402 |
| **Dominant hip BMD (g/cm^2^)** | 1.10 ± 0.13 | 1.06 ± 0.12 | 1.637 | 0.202 |
| **Dominant femoral neck BMD (g/cm^2^)** | 0.97 ± 0.15 | 0.94 ± 0.13 | 0.130 | 0.719 |
| **Nondominant leg BMD (g/cm^2^)** | 1.25 ± 0.09 | 1.26 ± 0.09 | 0.284 | 0.595 |
| **Nondominant hip BMD (g/cm^2^)** | 1.10 ± 0.13 | 1.07 ± 0.12 | 2.097 | 0.149 |
| **Nondominant femoral neck BMD (g/cm^2^)** | 0.96 ± 0.15 | 0.94 ± 0.14 | 0.061 | 0.805 |
| **Left arm BMD (g/cm^2^)** | 0.83 ± 0.06 | 0.84 ± 0.07 | 0.336 | 0.546 |
| **Right arm BMD (g/cm^2^)** | 0.86 ± 0.06 | 0.86 ± 0.07 | 0.052 | 0.819 |

Data are mean ± *SD*, WB - whole body, LS - lumbar spine, BMD - bone mineral density.

**Table 6.** Comparison bone mineral density of active runners in different running mileage group (<50 km/week and ≥50 km/week)

| **Variable** | **<50 (*n* *=* 280)** | **≥50 (*n* = 131)** | ***F*** | **Sig.** |
| --- | --- | --- | --- | --- |
| **WB BMD (g/cm^2^)** | 1.16 ± 0.08 | 1.17 ± 0.08 | 0.431 | 0.512 |
| **Spine BMD (g/cm^2^)** | 1.07 ± 0.13 | 1.05 ± 0.13 | 0.035 | 0.851 |
| **LS BMD (g/cm^2^)** | 1.05 ± 0.13 | 1.05 ± 0.13 | 0.041 | 0.839 |
| **Dominant leg BMD (g/cm^2^)** | 1.25 ± 0.09 | 1.28 ± 0.07 | 2.396 | 0.123 |
| **Dominant hip BMD (g/cm^2^)** | 1.08 ± 0.13 | 1.09 ± 0.10 | 2.344 | 0.127 |
| **Dominant femoral neck BMD (g/cm^2^)** | 0.96 ± 0.15 | 0.97 ± 0.13 | 3.355 | 0.068 |
| **Nondominant leg BMD (g/cm^2^)** | 1.25 ± 0.09 | 1.27 ± 0.08 | 1.318 | 0.252 |
| **Nondominant hip BMD (g/cm^2^)** | 1.09 ± 0.13 | 1.09 ± 0.12 | 1.157 | 0.283 |
| **Nondominant femoral neck BMD (g/cm^2^)** | 0.95 ± 0.15 | 0.96 ± 0.14 | 1.742 | 0.188 |
| **Left arm BMD (g/cm2)** | 0.84 ± 0.06 | 0.84 ± 0.07 | 0.080 | 0.777 |
| **Right arm BMD (g/cm2)** | 0.86 ± 0.06 | 0.86 ± 0.08 | 0.508 | 0.476 |

Data are mean ± *SD*, WB - whole body, LS - lumbar spine, BMD - bone mineral density.

However, to our knowledge, no unanimous beneficial threshold for BMD and mileage has been established as heterogenous samples were used and other factors such as genetics, nutrition, metabolic factors could play a significant role as well [6–9]. Based on analysis of our data (Supporting information) the mileage cut-off is likely greater than 105 km/week, which supports previous research that speculated it could be above the 100 km/week [3–5]. However, research has observed decreases in BMD in those running >32 km/week [1]. Further large sample studies with specific data on the amount of running are required before a more accurate threshold can be achieved.

References

1. MacDougall JD, Webber CE, Martin J, Ormerod S, Chesley A, Younglai E V., et al. Relationship among running mileage, bone density, and serum testosterone in male runners. J Appl Physiol. 1992;73: 1165–1170. doi:10.1152/jappl.1992.73.3.1165

2. Barrack MT, Fredericson M, Tenforde AS, Nattiv A. Evidence of a cumulative effect for risk factors predicting low bone mass among male adolescent athletes. Br J Sports Med. 2017;51: 200–205. doi:10.1136/bjsports-2016-096698

3. Bilanin JE, Blanchard MS, Russek-Cohen E. Lower vertebral bone density in male long distance runners. Med Sci Sports Exerc. 1989;21: 66–70. doi:10.1249/00005768-198902000-00012

4. Hetland ML, Haarbo J, Christiansen C. Low bone mass and high bone turnover in male long distance runners. J Clin Endocrinol Metab. 1993;77: 770–775. doi:10.1210/jcem.77.3.8370698

5. MacKelvie KJ. Bone mineral density and serum testosterone in chronically trained, high mileage 40-55 year old male runners. Br J Sports Med. 2000;34: 273–278. doi:10.1136/bjsm.34.4.273

6. Burrows M, Nevill AM, Bird S, Simpson D. Physiological factors associated with low bone mineral density in female endurance runners. Br J Sports Med. 2003;37: 67–71. doi:10.1136/bjsm.37.1.67

7. Hind K, Truscott JG, Evans JA. Low lumbar spine bone mineral density in both male and female endurance runners. Bone. 2006;39: 880–885. doi:10.1016/j.bone.2006.03.012

8. Kemmler W, Engelke K, Baumann H, Beeskow C, Stengel S, Weineck J, et al. Bone status in elite male runners. Eur J Appl Physiol. 2006;96: 78–85. doi:10.1007/s00421-005-0060-1

9. Tam N, Santos-Concejero J, Tucker R, Lamberts RP, Micklesfield LK. Bone health in elite Kenyan runners. Sports Medicine. 2018;36: 456–461. doi:10.1080/02640414.2017.1313998
