# Supplementary figures and images for: Comparison of bone mineral density of runners with inactive males: A cross-sectional 4HAIE study
Source: PLoS One. 2024 Aug 9;19(8):e0306715. doi: 10.1371/journal.pone.0306715 (PMC11315333; doi:10.1371/journal.pone.0306715)

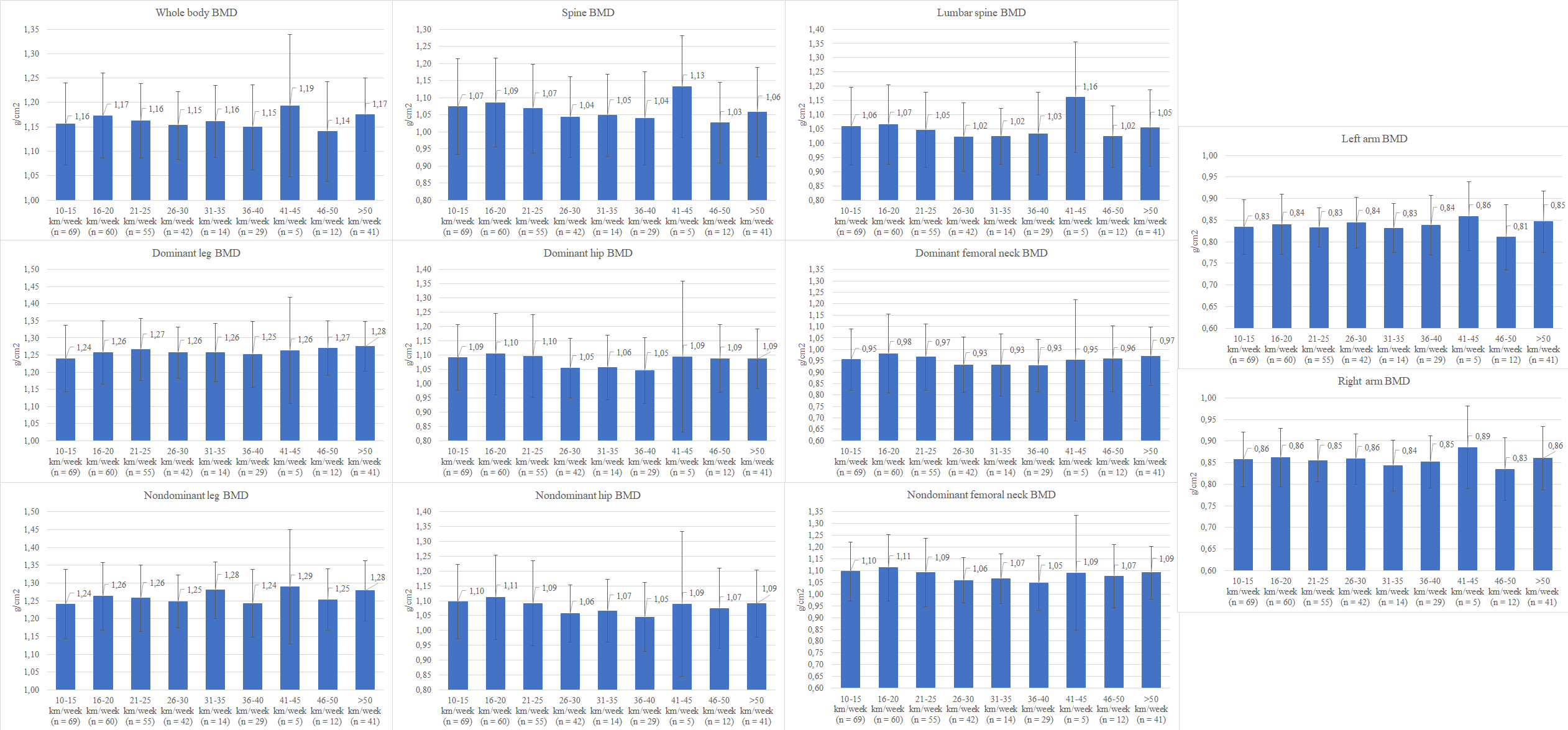

Supplement: S1 File — (ZIP) [file pone.0306715.s001.zip › KrajcigrFigure2.tiff]
