# Supplementary material for: Comparison of bone mineral density of runners with inactive males: A cross-sectional 4HAIE study
Source: PLoS One. 2024 Aug 9;19(8):e0306715. doi: 10.1371/journal.pone.0306715 (PMC11315333; doi:10.1371/journal.pone.0306715)
Supplement: Attachment — Submitted filename: Response to reviewers.docx [file pone.0306715.s002.docx]

Journal requirements:

When submitting your revision, we need you to address these additional requirements.

1. Please ensure that your manuscript meets PLOS ONE's style requirements, including those for file naming. The PLOS ONE style templates can be found at

https://journals.plos.org/plosone/s/file?id=wjVg/PLOSOne_formatting_sample_main_body.pdf and

<https://journals.plos.org/plosone/s/file?id=ba62/PLOSOne_formatting_sample_title_authors_affiliations.pdf>

Answer: Template was used for addressing the manuscript requirements. Additionally, the naming and style requirements have been read and followed.

2. We note that the grant information you provided in the ‘Funding Information’ and ‘Financial Disclosure’ sections do not match.

When you resubmit, please ensure that you provide the correct grant numbers for the awards you received for your study in the ‘Funding Information’ section.

Answer: Thank You for pointing out. We wanted to address this issue in revision and re-submission, however in the resubmitting process we were not able to change the statement in the ‘Financial Disclosure’ to ‘This research was funded by Program 4 HAIE—Healthy Aging in Industrial Environment (grant number: CZ.02.1.01/0.0/0.0/16_019/0000798). The funders had no role in study design, data collection and analysis, decision to publish, or preparation of the manuscript.’

3. Your ethics statement should only appear in the Methods section of your manuscript. If your ethics statement is written in any section besides the Methods, please delete it from any other section.

Answer: We have edited the text in the method section and deleted the statement from the other section.

4. We note that Figures 1 and 2 in your submission contain copyrighted images. All PLOS content is published under the Creative Commons Attribution License (CC BY 4.0), which means that the manuscript, images, and Supporting Information files will be freely available online, and any third party is permitted to access, download, copy, distribute, and use these materials in any way, even commercially, with proper attribution. For more information, see our copyright guidelines: http://journals.plos.org/plosone/s/licenses-and-copyright.

We require you to either (1) present written permission from the copyright holder to publish these figures specifically under the CC BY 4.0 license, or (2) remove the figures from your submission:

1. You may seek permission from the original copyright holder of Figures 1 and 2 to publish the content specifically under the CC BY 4.0 license.

We recommend that you contact the original copyright holder with the Content Permission Form (http://journals.plos.org/plosone/s/file?id=7c09/content-permission-form.pdf) and the following text:

“I request permission for the open-access journal PLOS ONE to publish XXX under the Creative Commons Attribution License (CCAL) CC BY 4.0 (http://creativecommons.org/licenses/by/4.0/). Please be aware that this license allows unrestricted use and distribution, even commercially, by third parties. Please reply and provide explicit written permission to publish XXX under a CC BY license and complete the attached form.”

Please upload the completed Content Permission Form or other proof of granted permissions as an "Other" file with your submission.

In the figure caption of the copyrighted figure, please include the following text: “Reprinted from [ref] under a CC BY license, with permission from [name of publisher], original copyright [original copyright year].”

2. If you are unable to obtain permission from the original copyright holder to publish these figures under the CC BY 4.0 license or if the copyright holder’s requirements are incompatible with the CC BY 4.0 license, please either i) remove the figure or ii) supply a replacement figure that complies with the CC BY 4.0 license. Please check copyright information on all replacement figures and update the figure caption with source information. If applicable, please specify in the figure caption text when a figure is similar but not identical to the original image and is therefore for illustrative purposes only.

Answer:

Figure 1 and 2 were taken from the Hologic Manual (2013) and modified to show the necessary information more clearly and concisely. A citation and reference have been added to the text.

However, if this is not a sufficient clarification and explanation, please let us know, we will address this issue differently.

Additional comments:

1. Thank you for confirming where you have obtained the images in Figure 1 and 2, and for including the corresponding citation in your manuscript.

Should your paper be accepted, all images will be published under PLOS’ Creative Commons Attribution (CC BY) 4.0 license, which means that they will be freely available online, and any third party is permitted to access, download, copy, distribute, and use these materials in any way, even commercially, with proper attribution. For more information, see our Figure guidelines: <http://www.plosone.org/static/figureGuidelines#policies>

With regards to the previously published and copyrighted figure in your submission, Figures 1 and 2, we require you to either present written permission from the copyright holder to publish this figure, or remove the figures from your submission. We have noted that the figure was published by the Hologic, Inc. and, therefore, ask that you please obtain permission from this company.

1. To seek permission from the Hologic, Inc. to publish Figures 1 and 2 under the specific Creative Commons Attribution License (CCAL), CC BY 4.0, please contact them with the following text:

“I request permission for the open-access journal PLOS ONE to publish XXX under the Creative Commons Attribution License (CCAL) CC BY 4.0 (<http://creativecommons.org/licenses/by/4.0/>). Please be aware that this license allows unrestricted use and distribution, even commercially, by third parties. Please reply and provide explicit written permission to publish XXX under a CC BY license.”

Please upload the granted permission to the manuscript as an other file. In the figure caption of the copyrighted figure, please include the following text: “Republished from [ref] under a CC BY license, with permission from [name of publisher], original copyright [original copyright year].”

Please note that RightsLink permission forms often impose use restrictions that are incompatible with our CC BY 4.0 license, and we are therefore unable to accept these permissions. For this reason, we strongly recommend contacting copyright holders with the PLOS ONE Request for Permission form.

2. If you are unable to obtain permission from the journal, please either A) remove the figure or B) supply a replacement figure that complies with the CC BY 4.0 license. Please check copyright information on all replacement figures and update the figure caption with source information. If applicable, please specify in the figure caption text when a figure is similar but not identical to the original image used in the study, and is therefore for illustrative purposes only.

Additional answer:

Thank you for clarifying the possible solutions to this problem. After careful consideration and taking into account many circumstances, we have decided to remove Figures 1 and 2 from the text and leave only the reference to the relevant manual. Based on these changes, we have also adjusted the numbering of the retained Figures.

Additional Editor Comments:

In the methods section of the abstract, provide a summary of study site, how participants were recruited. Also indicate that these were male runners. Mention the partial body segments where BMD was measured.

In the results section of the abstract, provide figures in brackets to back the statistical significance of the comparisons. Provide a summary of results of the pairwise comparison for AR and INC in the same age group. Figures are important to the reader to verify the differences.
Answer:

We edited the text. Information about the recruitment, sex and measured body segments were added.

In the result section we provided figures in brackets. Additionally, we provided the pairwise comparison.

Reviewers' comments:

Reviewer's Responses to Questions

**Comments to the Author**

1. Is the manuscript technically sound, and do the data support the conclusions?

The manuscript must describe a technically sound piece of scientific research with data that supports the conclusions. Experiments must have been conducted rigorously, with appropriate controls, replication, and sample sizes. The conclusions must be drawn appropriately based on the data presented.

Reviewer #1: Yes

2. Has the statistical analysis been performed appropriately and rigorously?

Reviewer #1: Yes

3. Have the authors made all data underlying the findings in their manuscript fully available?

The [PLOS Data policy](http://www.plosone.org/static/policies.action#sharing) requires authors to make all data underlying the findings described in their manuscript fully available without restriction, with rare exception (please refer to the Data Availability Statement in the manuscript PDF file). The data should be provided as part of the manuscript or its supporting information, or deposited to a public repository. For example, in addition to summary statistics, the data points behind means, medians and variance measures should be available. If there are restrictions on publicly sharing data—e.g. participant privacy or use of data from a third party—those must be specified.

Reviewer #1: Yes

4. Is the manuscript presented in an intelligible fashion and written in standard English?

PLOS ONE does not copyedit accepted manuscripts, so the language in submitted articles must be clear, correct, and unambiguous. Any typographical or grammatical errors should be corrected at revision, so please note any specific errors here.

Reviewer #1: No

Answer:

We used the academic English language tool Writefull. Moreover, a native English speaker from the kinanthropology academic field has revised the manuscript.

5. Review Comments to the Author

Please use the space provided to explain your answers to the questions above. You may also include additional comments for the author, including concerns about dual publication, research ethics, or publication ethics. (Please upload your review as an attachment if it exceeds 20,000 characters)

Reviewer #1: Thank you for giving me the opportunity to review this paper. It is interesting and much work has gone into its preparation, however, there are multiple grammatical errors and clarity problems (for example first sentence on page 4). There are words missing (e.g., page 4, starting with "Furthermore...") and incomplete sentences (e.g., in last paragraph page 20, starting with "compared with..."

Answer: Thank You for the kind words and moreover thank You for addressing the issues of this manuscript. To address grammatical errors and clarity problems we revised the whole text. Additionally, we used the academic English language tool Writefull. Moreover, a native English speaker from the kinanthropology academic field has revised the manuscript.

In my opinion there are several issues that need to be addressed before the manuscript can get considered for publication:
1. the conclusion that 'running is good for BMD' has been discussed before, so the novelty is not there. However, is it possible that too much running is NOT good for the BMD of the spine? The paper does not discuss how much running is good (and the amount of running was not measured). I believe that the literature has been trying to establish that there is a limit after which running becomes unfavorable to spine BMD.

Answer:

Thank You for this point.

We are aware of the fact, that some studies indicated that too much running may not be beneficial for bone health. E.g., Burrows et al. (2003) showed that greater running mileage was negatively associated with lumbar spine and femoral neck BMD. Additionally, weekly running distance was inversely associated lumbar spine BMD in the study of Hind et al. (2006). The concern for lumbar spine as a site prone to low BMD was in some other studies (Barrack et al., 2008; Bilanin et al., 1989; Fredericson et al., 2007; Goodpaster et al., 1996; Hetland et al., 1993; MacDougall et al., 1992; MacKelvie, 2000; Tam et al., 2018; Winters et al., 1996)

Greater BMD values for runners in more loaded sites (i.e., legs, calcaneus hip, Wards triangle, trochanter, and femoral neck) has been shown previously (Aloia et al., 1978; Brahm et al., 1997; Fredericson et al., 2007; Heinonen et al., 1993; Kohrt et al., 2004; Piasecki et al., 2018; Tam et al., 2018; Wolman et al., 1991). Likewise lower values for lumbar spine have been seen previously (Bilanin et al., 1989; Brahm et al., 1997; Fredericson et al., 2007; Goodpaster et al., 1996; Hetland et al., 1993; Tam et al., 2018). However, one study has shown greater BMD for lumbar spine (Lane et al., 1998).

Hind and colleagues (Hind et al., 2006) aimed to address the possible explanation for the difference in lumbar spine values compared to other sites. The reason may be due the lumbar spine as predominantly trabecular bone which may be less influenced by lower body impact loading and local muscle action.

Regarding the beneficial running mileage threshold:

To our knowledge, no unanimous threshold for mileage has been established as heterogenous samples were used and other factors such as genetics, nutrition, metabolic factors etc. could play a significant role as well (Burrows et al., 2003; Hind et al., 2006; Kemmler et al., 2006; Tam et al., 2018).

Some studies indicated that the threshold for proximal femur could exist, and it could be somewhere between 90-100 km/week (Bilanin et al., 1989; Hetland et al., 1993; MacKelvie, 2000).

Regarding the lumbar spine, some studies found lower vertebral BMD in young adult runners whose training over ≈ 92 km/ week in compare with controls (Bilanin et al., 1989; Hetland et al., 1993).

From those studies that were trying to focus on the mileage:

Hetland et al. (1993), whose compared elite runners and controls, found out that the difference increased with the weakly distance. Those who ran more than 100 km/week had the lumbar bone mineral content, on the average, 19% lower. Moreover, Lumbar BMC was negatively correlated with the weekly distance run (r = -0.37; P < 0.0001), with a difference of 19 +/- 5% (mean +/- SEM). A similar relation was also found for all measurement sites.

MacDougal et al. (1992) studied controls and runners in 5 mileage groups. The groups were as follows: controls, 5-10 miles/week, 15-20 miles/week, 25-30 miles/week, 40-55 miles/week, 60-75 miles/week.

They found no statistically significant difference in BMD of spine and trunk. However, BMD of lower legs was increasing from controls and 5-10 miles/week group to 15-20 miles/week group, where the values were the greatest. After this peak, decrease was showed thorough the 25-30 miles/week, 40-55 miles/week, and 60-75 miles/week groups.

Kemmler et al. (2006) observed a low, but not signiﬁcant association between training volume (km/week/year) and trabecular BMD of the femoral neck. This association disappeared when adjusting for age, BMI, and body fat in the group of highly trained male runners. They postulated that: “The eﬀect of long distance running per se on bone parameters is not deleterious.”

Barrack and colleagues (2017) identified risk factors for low BMD: running >30 miles/week was one of the strongest risk factors predicting low BMD of lumbar spine, however it was not for WB.

(Hind et al., 2006) presented in their manuscript that there were moderate negative correlations between weekly running distance and LS BMD (r2 = 0.267; 0.189; P < 0.001).

We were also considering adding this aim and information to the manuscript. However, we elected not to include it as the purpose of this study is different. Adequate information has been used and added to the introduction and discussion section.

Based on the aforementioned studies, we might presume that the threshold could be somewhere about 90-100 km/week. But as it is written above, to our knowledge, not a unanimous threshold for mileage has been established. If You are referring to a specific study, we would like to ask You for a reference to the research mentioned.

Partial analysis has been performed (Supporting information) to provide additional data on the beneficial threshold of running and BMD. For this analysis we used information about running mileage and frequency from the surveys.

Three partial analyses were performed. For the first analysis the runners were divided into 9 sub-groups (inspired by (MacDougall et al., 1992)): 10-15 km/week, 16-20 km/week, 21-25 km/week, 26-30 km/week, 31-35km/week, 36-40 km/week, 41-45 km/week, 46-50 km/week, ≥51 km/week. Additionally, a pairwise comparison of different running mileage groups was conducted. Results of this analysis can be seen in Figure 2. For the second analysis the runners were divided into 2 sub-groups: <30 km/ week and ≥30 km/week. For the third analysis (based on the findings from (Barrack et al., 2017)) the runners were divided into 2 sub-groups: <50 km/ week and ≥50 km/week. To compare BMD of AR in different running mileage groups and eliminate confounding variables, ANCOVA with Bonferroni correction was used in all analyses. Confounding variables for all analyses were age, mass, height, BMI, fat mass, and lean mass. All three analyses found no significant difference in BMD between groups at every measured site (p>0.05). Results of the second and third analysis can be seen in Table 5 and 6, respectively.

These analyses were the only three methods of group separation we deemed reasonable. Higher mileage (>90-100 km/week) as stated by previous studies (Bilanin et al., 1989; Hetland et al., 1993; MacKelvie, 2000) groups were not possible to use and compare.

However, those results may indicate that there is no threshold in range of mileage used the analysis (10-105 km/week). But based on the samples in partial mileage groups and their distribution we cannot consider these results to be reliable. The information about the running mileage and frequency were based on survey data, which is a limitation for performing these analyses. for this case, where we would need more precise values to be able sort out the runners, is big limitation. Moreover, we believe that the design for this type of study would be different.

To address this issue Supporting information is provided and text in the discussion section of the manuscript is included:

Based on analysis of our data (Supporting information) the mileage cut-off is likely greater than 105 km/week (65 miles/week), which supports previous research that speculated it could be above the 100 km/week (62 miles/week) [35,37,52]. However, research has observed decreases in BMD in those running >20 miles/week [53]. Further large sample studies with specific data on the amount of running are required before a more accurate threshold can be achieved.

2. A major weakness of this study was the criterion that was chosen to be considered 'active' vs. non-active. 'Running for 6 weeks (or less) prior to data collection' is not a good discriminator between "active" and non-active participants. This short amount of time might have consequences to the cardiovascular system, but I doubt that it could affect BMD

Answer:

Thank You for pointing out this issue.

6 weeks prior to the study was inclusion minimum. Participants had been running for at least 1 year prior to measurement, which is sufficient for bone remodelling to take place (Hadjidakis & Androulakis, 2006)

The text in the introduction section was edited and the information about remodelling process was added.

We also edited the text in the method section (subjects): AR must have reported meeting WHO public PA guidelines [36] (150 min/week, moderate or 75 min/vigorous PA, or a combination of both) and running a minimum of 10 km per week for at least one year prior to the study.

3. BMD of the right leg vs. left leg was compared, it should have been 'dominant' vs. 'non-dominant' leg

Thank you for your comment. It was possible to address this issue.

We used additional information from the 4HAIE study - functional testing to assed the lower limb dominance. Based on the Hoffman et al. (1998) the dominance of leg “ball kick” test was used. However, some participants must have been excluded from the study due to the missing data from this test.

Based on the results of the “ball kick” test we assigned the values for dominant and nondominant leg. Furthermore, we used the dominant and nondominant division for hip and femoral neck as well.

From all the participants (*n* = 539) included in the revised study 515 participants had the right leg as their dominant leg and 24 participants had the left leg as their dominant leg. Additionally, 16 out of 327 in the AR group were left leg dominant and 8 out of 212 in the INC group were left leg dominant.

We have performed and included in the manuscript the statistical analysis for BMD of dominant and nondominant side instead of left and right side for the leg, hip and femoral neck.

4. page 21: the authors discuss the 'unloading' of the spine, but at the same time explain how the loading or 'shock' wave travels through the spine. I don't believe that the spine is 'unloaded', but it is indeed loaded during running. There are many references in the literature that support this notion.

Answer:

Thank you for this comment and pointing out this issue.

In the discussion section we edited the text and change the expression from spine and lumbar spine as the sites experiencing less load. This statement is based on the statement which is written in the introduction section: “The shock wave, which occurs in running, is initiated by contact of the runners’ feet with the ground and is transmitted by the skeletal system to the head of the runner.” (Gruber et al., 2014; Hamill et al., 1995)

We also addressed the possible explanation for the differences of the values of lumbar spine compared to other sites in the discussion section of the manuscript.

5. "Mitchell" is spelled with 2 'l'

Answer: Thank You for pointing out this issue. It is now spelled with 2 ‘l’s.

6. The inclusion of hundreds of participants is definitely a strength of this study, but it gets weakened by lack of variables that were controlled, e.g. running speed, running time, days and miles/km per week, etc. and the amount of body areas that were compared. I believe that one or two focused area (for example the lumbar spine, since this is the only area that has been the point of discussion in terms of BMD) would have made this study stronger.

Answer:

Thank You for this comment.

To address the running variables issue:

Based on the available information we calculated the average pace for AR. We added this information to the manuscript. It can be seen in method section (subjects) of the manuscript: In total, it is on average 29.69 ± 18.87 km/week and 175.00 ± 114.26 min/week with average pace 6.06 ± 1.72 min/km.

Unfortunately, precise information about the time (years) runners had been running is not possible due to the categorical answers. To the question: "How long have you been running?" Participants could choose one of the following answers: (1) I plan to start (2) less than 0.5 years (3) 1-2 years (4) 2-5 years (5) 5-8 years (6) 8 or more years (7) I do not run

Comparison of runners in different pace groups (similar to the partial mileage analysis mentioned above in the comment 1) was performed. The difference of BMD in measured sites was not statically significant. To compare BMD of AR of different pace and eliminate confounding variables, ANCOVA with Bonferroni correction was used. The dependent variables were BMD of whole-body (WB), spine, lumbar spine (LS), dominant leg, dominant hip, dominant femoral neck, nondominant leg, nondominant hip BMD, nondominant femoral neck, left arm, and right arm. For ANCOVA analyses the controlling variables were age, mass, height, BMI, fat mass and lean mass.

Those results may indicate that there is no threshold in range of pace used in the analysis. However, based on the sample size in partial pace groups and their distribution we cannot consider these results to be reliable. The information was based on survey data, and we would need more precise values to make more accurate analyses, which is a big limitation. Moreover, we believe that the design for a study of this nature would be different to the current study. The design of the project 4HAIE, from which the data was taken, was not focused in this direction, so it is unfortunately not possible for the article to focus on this problem in detail.

To address the compared body parts issue:

Areas of body that has been compared (WB, spine, lumbar spine, leg, hip, femoral neck, arm).

We used whole body, legs as the most likely affected segment by running (Gruber et al., 2014; Hamill et al., 1995), lumbar spine, hip and femoral neck as they are used for diagnosis of osteoporosis in clinical medicine (Cosman et al., 2014; Lane, 2006) or were shown in previous studies as sites with heterogenous values (Barrack et al., 2017; Bennell et al., 1997; Bilanin et al., 1989; Brahm et al., 1997; Hetland et al., 1993; Kemmler et al., 2006; MacDougall et al., 1992).

Additionally, we have added information about different segments in the introduction and discussion section.

6. PLOS authors have the option to publish the peer review history of their article ([what does this mean?](https://journals.plos.org/plosone/s/editorial-and-peer-review-process#loc-peer-review-history)). If published, this will include your full peer review and any attached files.


If you choose “no”, your identity will remain anonymous but your review may still be made public.


**Do you want your identity to be public for this peer review?** For information about this choice, including consent withdrawal, please see our [Privacy Policy](https://www.plos.org/privacy-policy).

Reviewer #1:

References

Aloia, J. F., Cohn, S. H., Babu, T., Abesamis, C., Kalici, N., & Ellis, K. (1978). Skeletal mass and body composition in marathon runners. *Metabolism*, *27*(12), 1793–1796. https://doi.org/10.1016/0026-0495(78)90265-2

Barrack, M. T., Fredericson, M., Tenforde, A. S., & Nattiv, A. (2017). Evidence of a cumulative effect for risk factors predicting low bone mass among male adolescent athletes. *British Journal of Sports Medicine*, *51*(3), 200–205. https://doi.org/10.1136/bjsports-2016-096698

Barrack, M. T., Rauh, M. J., Barkai, H. S., & Nichols, J. F. (2008). Dietary restraint and low bone mass in female adolescent endurance runners. *American Journal of Clinical Nutrition*, *87*(1), 36–43. https://doi.org/10.1093/ajcn/87.1.36

Bennell, K. L., Malcolm, S. A., Khan, K. M., Thomas, S. A., Reid, S. J., Brukner, P. D., Ebeling, P. R., & Wark, J. D. (1997). Bone mass and bone turnover in power athletes, endurance athletes, and controls: A 12-month longitudinal study. *Bone*, *20*(5), 477–484. https://doi.org/10.1016/S8756-3282(97)00026-4

Bilanin, J. E., Blanchard, M. S., & Russek-Cohen, E. (1989). Lower vertebral bone density in male long distance runners. *Medicine & Science in Sports & Exercise*, *21*(1), 66–70. https://doi.org/10.1249/00005768-198902000-00012

Brahm, H., Ström, H., Piehl-Aulin, K., Mallmin, H., & Ljunghall, S. (1997). Bone metabolism in endurance trained athletes: A comparison to population-based controls based on DXA, SXA, quantitative ultrasound, and biochemical markers. *Calcified Tissue International*, *61*(6), 448–454. https://doi.org/10.1007/s002239900366

Burrows, M., Nevill, A. M., Bird, S., & Simpson, D. (2003). Physiological factors associated with low bone mineral density in female endurance runners. *British Journal of Sports Medicine*, *37*(1), 67–71. https://doi.org/10.1136/bjsm.37.1.67

Cosman, F., de Beur, S. J., LeBoff, M. S., Lewiecki, E. M., Tanner, B., Randall, S., & Lindsay, R. (2014). Clinician’s Guide to Prevention and Treatment of Osteoporosis. *Osteoporosis International*, *25*(10), 2359–2381. https://doi.org/10.1007/s00198-014-2794-2

Fredericson, M., Chew, K., Ngo, J., Cleek, T., Kiratli, J., & Cobb, K. (2007). Regional bone mineral density in male athletes: A comparison of soccer players, runners and controls. *British Journal of Sports Medicine*, *41*(10), 664–668. https://doi.org/10.1136/bjsm.2006.030783

Goodpaster, B. H., Costill, D. L., Trappe, S. W., & Hughes, G. M. (1996). The relationship of sustained exercise training and bone mineral density in aging male runners. *Scandinavian Journal of Medicine & Science in Sports*, *6*(4), 216–221. https://doi.org/10.1111/j.1600-0838.1996.tb00094.x

Gruber, A. H., Boyer, K. A., Derrick, T. R., & Hamill, J. (2014). Impact shock frequency components and attenuation in rearfoot and forefoot running. *Journal of Sport and Health Science*, *3*(2), 113–121. https://doi.org/10.1016/j.jshs.2014.03.004

Hadjidakis, D. J., & Androulakis, I. I. (2006). Bone Remodeling. *Annals of the New York Academy of Sciences*, *1092*(1), 385–396. https://doi.org/10.1196/annals.1365.035

Hamill, J., Derrick, T. R., & Holt, K. G. (1995). Shock attenuation and stride frequency during running. *Human Movement Science*, *14*(1), 45–60. https://doi.org/10.1016/0167-9457(95)00004-C

Heinonen, A., Oja, P., Kannus, P., Sievänen, H., Mänttäri, A., & Vuori, I. (1993). Bone mineral density of female athletes in different sports. *Bone and Mineral*, *23*(1), 1–14. https://doi.org/10.1016/S0169-6009(08)80086-4

Hetland, M. L., Haarbo, J., & Christiansen, C. (1993). Low bone mass and high bone turnover in male long distance runners. *The Journal of Clinical Endocrinology & Metabolism*, *77*(3), 770–775. https://doi.org/10.1210/jcem.77.3.8370698

Hind, K., Truscott, J. G., & Evans, J. A. (2006). Low lumbar spine bone mineral density in both male and female endurance runners. *Bone*, *39*(4), 880–885. https://doi.org/10.1016/j.bone.2006.03.012

Hoffman, M., Schrader, J., Applegate, T., & Koceja, D. (1998). Unilateral postural control of the functionally dominant and nondominant extremities of healthy subjects. *Journal of Athletic Training*, *33*(4), 319–322.

*Hologic. QDR for Windows XP Reference Manual. Hologic Inc.* (2013).

Kemmler, W., Engelke, K., Baumann, H., Beeskow, C., Stengel, S., Weineck, J., & Kalender, W. A. (2006). Bone status in elite male runners. *European Journal of Applied Physiology*, *96*(1), 78–85. https://doi.org/10.1007/s00421-005-0060-1

Kohrt, W. M., Bloomfield, S. A., Little, K. D., Nelson, M. E., & Yingling, V. R. (2004). Physical activity and bone health. *Medicine and Science in Sports and Exercise*, *36*(11), 1985–1996. https://doi.org/10.1249/01.MSS.0000142662.21767.58

Lane, N. E. (2006). Epidemiology, etiology, and diagnosis of osteoporosis. *American Journal of Obstetrics and Gynecology*, *194*(2 SUPPL.). https://doi.org/10.1016/j.ajog.2005.08.047

Lane, N. E., Oehlert, J. W., Bloch, D. A., & Fries, J. F. (1998). The relationship of running to osteoarthritis of the knee and hip and bone mineral density of the lumbar spine: a 9 year longitudinal study. *The Journal of Rheumatology*, *25*(2), 334–341.

MacDougall, J. D., Webber, C. E., Martin, J., Ormerod, S., Chesley, A., Younglai, E. V., Gordon, C. L., & Blimkie, C. J. (1992). Relationship among running mileage, bone density, and serum testosterone in male runners. *Journal of Applied Physiology*, *73*(3), 1165–1170. https://doi.org/10.1152/jappl.1992.73.3.1165

MacKelvie, K. J. (2000). Bone mineral density and serum testosterone in chronically trained, high mileage 40-55 year old male runners. *British Journal of Sports Medicine*, *34*(4), 273–278. https://doi.org/10.1136/bjsm.34.4.273

Piasecki, J., McPhee, J. S., Hannam, K., Deere, K. C., Elhakeem, A., Piasecki, M., Degens, H., Tobias, J. H., & Ireland, A. (2018). Hip and spine bone mineral density are greater in master sprinters, but not endurance runners compared with non-athletic controls. *Archives of Osteoporosis*, *13*(1). https://doi.org/10.1007/s11657-018-0486-9

Tam, N., Santos-Concejero, J., Tucker, R., Lamberts, R. P., & Micklesfield, L. K. (2018). Bone health in elite Kenyan runners. *Sports Medicine*, *36*(4), 456–461. https://doi.org/10.1080/02640414.2017.1313998

Winters, K. M., Adams, W. C., Meredith, C. N., Van Loan, M. D., & Lasley, B. L. (1996). Bone density and cyclic ovarian function in trained runners and active controls. *Medicine &amp Science in Sports &amp Exercise*, *28*(7), 776–785. https://doi.org/10.1097/00005768-199607000-00002

Wolman, R. L., Faulmann, L., Clark, P., Hesp, R., & Harries, M. G. (1991). Different training patterns and bone mineral density of the femoral shaft in elite, female athletes. *Annals of the Rheumatic Diseases*, *50*(7), 487–489. https://doi.org/10.1136/ard.50.7.487
