# Supplementary material for: Comparison of bone mineral density of runners with inactive males: A cross-sectional 4HAIE study
Source: PLoS One. 2024 Aug 9;19(8):e0306715. doi: 10.1371/journal.pone.0306715 (PMC11315333; doi:10.1371/journal.pone.0306715)
Supplement: Attachment — Submitted filename: Response to Reviewers.docx [file pone.0306715.s003.docx]

Reviewers' comments:

Reviewer's Responses to Questions

**Comments to the Author**

1. If the authors have adequately addressed your comments raised in a previous round of review and you feel that this manuscript is now acceptable for publication, you may indicate that here to bypass the “Comments to the Author” section, enter your conflict of interest statement in the “Confidential to Editor” section, and submit your "Accept" recommendation.

Reviewer #2: (No Response)

2. Is the manuscript technically sound, and do the data support the conclusions?

The manuscript must describe a technically sound piece of scientific research with data that supports the conclusions. Experiments must have been conducted rigorously, with appropriate controls, replication, and sample sizes. The conclusions must be drawn appropriately based on the data presented.

Reviewer #2: Partly

3. Has the statistical analysis been performed appropriately and rigorously?

Reviewer #2: Yes

4. Have the authors made all data underlying the findings in their manuscript fully available?

The [PLOS Data policy](http://www.plosone.org/static/policies.action#sharing) requires authors to make all data underlying the findings described in their manuscript fully available without restriction, with rare exception (please refer to the Data Availability Statement in the manuscript PDF file). The data should be provided as part of the manuscript or its supporting information, or deposited to a public repository. For example, in addition to summary statistics, the data points behind means, medians and variance measures should be available. If there are restrictions on publicly sharing data—e.g. participant privacy or use of data from a third party—those must be specified.

Reviewer #2: Yes

5. Is the manuscript presented in an intelligible fashion and written in standard English?

PLOS ONE does not copyedit accepted manuscripts, so the language in submitted articles must be clear, correct, and unambiguous. Any typographical or grammatical errors should be corrected at revision, so please note any specific errors here.

Reviewer #2: Yes

6. Review Comments to the Author

Please use the space provided to explain your answers to the questions above. You may also include additional comments for the author, including concerns about dual publication, research ethics, or publication ethics. (Please upload your review as an attachment if it exceeds 20,000 characters)

Reviewer #2: Although the authors achieve the established objective, there are a set of variables that remain unexplained. Food frequency or other variables (strength training, professional activity) that may have implications for BMD stand out.
The fact that we are dealing with a "large" sample cannot be ignored, on the other hand there are many variables that remain uncontrolled.
The limitations should be clear that they were not controlled and that they could have implications on the results.

Thank you for your comment and for highlighting additional variables that were not clearly stated. We have edited the text in the ‘Strengths and limitations’ section to include these variables. Unfortunately, we were not able to use these variables in the analyses because the data for this paper were taken from the 4HAIE project. The design of the 4HAIE project was not focused specifically in this direction and the variables you have specified were not evaluated in the 4HAIE study. On the other hand, the study accounted for other variables that could be used from the 4HAIE project and have been previously shown to be associated with BMD such as age, mass, height, BMI, fat mass and lean mass [11,22,23,26–29,49]. Additionally, because it was not possible to control for all variables affecting (potentially affecting) BMD, control segments (upper limbs) were used.

Specifically:
In the Strength and limitations section we have added additional information to the sentence that refers to nutrition to further specify nutrition.

“Moreover, the study lacked information on other important determinants of BMD, such as childhood history of PA, nutrition **(such as diet, food frequency etc.),** or genetics.”

We have added a sentence to the limitations about variables that were not controlled and about possible bias in the results based on the absence of those variables.

“Furthermore, other PA that may influence BMD such as strength training, aerobic training, and individual or team sport participation (including level of expertise) were not controlled in the analyses and could have impacted the results seen [22,26,32,33,56–59].”

We have also added information about the variables that were controlled to the Strength and limitations section so that the information about what was and was not controlled is as accurate as possible.

“On the other hand, the analysis was controlled for variables (age, mass, height, BMI, fat mass and lean mass) found to be associated with BMD in previous research [11,22,23,26–29,49].”

The tables need to be improved; they are difficult to read, particularly the 4.

Thank you for this comment. For all tables, we have adjusted the centred text alignment for the result values and their names to make them easier to read. The captions under all tables have been reviewed and adjusted to ensure they are as clear and comprehensive as possible, in conjunction with the labels within the tables themselves. Moreover, we focused on the most problematic tables separately.

Regarding Table 1, the numbers of subjects have been removed, as these are provided in the methodology section. Additionally, a more detailed description of the values in the table has been added (M **±** SD, “*d”* added to Effect size).

Regarding Table 2, the numbers of subjects have been removed, as these are provided in the methodology section. Additionally, a more detailed description of the values in the table has been added.

Regarding Table 3: we have changed the orientation of the page to make the table more readable and to enable increasing the size of the font. We have added information indicating that the segments represent "differences and *p* values” in the table to enhance clarity when reading the table.

Regarding Table 4: we have divided this table into two tables. Specifically, into upper body and lower body. We further separated it from the text. Moreover, we did some additional formatting in the tables (spaces, borders, …).

7. PLOS authors have the option to publish the peer review history of their article ([what does this mean?](https://journals.plos.org/plosone/s/editorial-and-peer-review-process#loc-peer-review-history)). If published, this will include your full peer review and any attached files.


If you choose “no”, your identity will remain anonymous but your review may still be made public.


**Do you want your identity to be public for this peer review?** For information about this choice, including consent withdrawal, please see our [Privacy Policy](https://www.plos.org/privacy-policy).

Reviewer #2: No

[NOTE: If reviewer comments were submitted as an attachment file, they will be attached to this email and accessible via the submission site. Please log into your account, locate the manuscript record, and check for the action link "View Attachments". If this link does not appear, there are no attachment files.]

While revising your submission, please upload your figure files to the Preflight Analysis and Conversion Engine (PACE) digital diagnostic tool, <https://pacev2.apexcovantage.com/>. PACE helps ensure that figures meet PLOS requirements. To use PACE, you must first register as a user. Registration is free. Then, login and navigate to the UPLOAD tab, where you will find detailed instructions on how to use the tool. If you encounter any issues or have any questions when using PACE, please email PLOS at [figures@plos.org](mailto:figures@plos.org). Please note that Supporting Information files do not need this step.

*In compliance with data protection regulations, you may request that we remove your personal registration details at any time.* [*(Remove my information/details)*](https://www.editorialmanager.com/pone/login.asp?a=r)*. Please contact the publication office if you have any questions.*
